# Multisystem Mitochondrial Disease Associated With a Mare m.10000G>A Mitochondrial tRNA^**Gly**^ (MT-TG) Variant

**DOI:** 10.3389/fneur.2022.795060

**Published:** 2022-03-30

**Authors:** Haiyan Yang, Victor Wei Zhang, Liang Ai, Siyi Gan, Liwen Wu

**Affiliations:** ^1^Neurology Department, Hunan Children's Hospital, The School of Pediatrics, Hengyang Medical School, University of South China, Changsha, China; ^2^Department of Human and Molecular Genetics, Baylor College of Medicine, Houston, TX, United States

**Keywords:** mitochondrial disease, m.10000G>A, mitochondrial tRNA Gly, children, neurology—clinical

## Abstract

**Background:**

Mitochondrial diseases are clinically heterogeneous, can occur at any age, and can manifest with a wide range of clinical symptoms. They can involve any organ or tissue, characteristically involve multiple systems, typically affecting organs that are highly dependent on aerobic metabolism, and making a definitive molecular diagnosis of a mitochondrial disorder is challenging.

**Methods:**

Clinical data of the proband and his family members were gathered in a retrospective study. Whole-exome sequencing and full-length sequencing of the mitochondrial genome that were performed on peripheral blood, urine, and oral mucosa cells were applied for genetic analysis.

**Results:**

In this study, we reported a childhood-onset mitochondrial phenotype in a 13-year-old patient. Analysis of the next-generation sequencing data of the nuclear genome and the full-length sequencing of the mitochondrial genome revealed the rare m.10000G>A variant in MT-TG that was present at variable heteroplasmy levels across tissue types: 32.7% in the blood, 56.15% in urinary epithelial cells, and 27.3% in oral mucosa cells. No variant was found in the peripheral blood of his mother and sister. No pathogenic mutation of nDNA was found.

**Conclusion:**

Our results added evidence that the *de novo* m.10000G>A variation in the highly conserved sequence of MT-TG appears to suggest a childhood-onset mitochondrial phenotype in the 13-year-old patient, thus broadening the genotypic interpretation of mitochondrial DNA-related diseases.

## Background

Mitochondrial diseases are a group of genetic disorders that are characterized by defects in oxidative phosphorylation and are caused by mutations in genes in the nuclear DNA (nDNA) and mitochondrial DNA (mtDNA) that encode structural mitochondrial proteins or proteins involved in mitochondrial function (1). Mt-tRNA genes account for only ~8% of the entire mitochondrial genome (2). At present, there are only 11 MT-TG variants reported in MITOMAP^1^,^1^ (see Table 1); among them, only 1 variant, m.10010T>C, is considered “definitely pathogenic”, and 2 variants, m.9997T>C and m.9997T>A, are considered “likely pathogenic.” m.10010T>C was reported 11 times before, and is defined as a pathogenic variant that may lead to severe mitochondrial encephalomyopathy, seizures, choreoathetoid movements, progressive ataxia, and spastic tetraparesis, and results in clinical phenotypes such as rahbdomyolysis, myoglobinuria, diffuse myalgias, muscular cramps, limb weakness, lower limb neuropathy, and hypothyroidism (3–5). m.9997T>A was not reported in the literature to have corresponding clinical phenotypes. A clinical phenotype of m.9997T>C reported in the literature was maternally inherited hypertrophic cardiomyopathy (MHCM) (6) (see Table 1 for details). In this study, we reported an unreported MT-TG variant, m.10000G>A, in the highly conserved sequence of MT-TG that appears to suggest a childhood-onset mitochondrial phenotype in a 13-year-old patient, thus broadening the genotypic interpretation of mtDNA-related diseases.

**Table 1:** 11 MT-TG variants reported in MITOMAP.

**MT-TG variants**	**Disease**	**Allele**	**RNA**	**Homo/He** **terplasmy**	**Status**	**MitoTIP**
10005	Hearing loss patient	A10005G	tRNA Gly	Nr/nr	Reported	9.00%
10006	CIPO/ Encephalopathy	A10006G	tRNA Gly	±	Unclear	19.30%
10044	SIDS	A10044G	tRNA Gly	±	Unclear	34.70%
10055	Tic disorder patient/hearing loss patient	A10055G	tRNA Gly	Nr/nr	Reported	29.70%
10019	Hearing loss patient	C10019T	tRNA Gly	Nr/nr	Reported	50.50%
10014	Myopathy	G10014A	tRNA Gly	±	Unclear	60.90%
10003	Hypertension/ maternally inherited diabetes/hearing loss	T10003C	tRNA Gly	±	Reported	0.40%
10010	PEM	T10010C	tRNA Gly	±	Cfrm	Pathogenic
10057	Hearing loss patient	T10057C	tRNA Gly	Nr/nr	Reported	38.40%
9997	Unspecified patient from clinical lab	T9997A	tRNA Gly	Nr/nr	Reported	95.20%
9997	MHCM	T9997C	tRNA Gly	±	Reported	80.30%

*CIPO, chronic intestinal pseudoobstruction with myopathy and ophthalmoplegia; PEM, progressive encephalopathy; SIDS, sudden infant death syndrome; MHCM, maternally inherited hypertrophic cardiomyopathy*.

## Methods

### Patient

One patient was included in the study. The patient was managed at the Department of Neurology, Hunan Children's Hospital. The parents of the patients provided written informed consent. This study was approved by the Medical Ethics Committee of Hunan Children's Hospital.

### Whole-Exome Sequencing of Peripheral Blood

The whole-exome sequencing method was performed according to our previous research methods (7). Sequence variants were annotated using population and literature databases such as 1,000 Genomes, dbSNP, GnomAD^2^, Clinvar^3^, HGMD, and OMIM^4^. Variant interpretation was performed according to the American College of Medical Genetics (ACMG) guidelines (8). Whole-exome sequencing was performed simultaneously on samples from the patient, his parents, and the elder sister.

### Full-Length Sequencing of the Mitochondrial Genome

Full-length sequencing of the mitochondrial genome of peripheral blood, urine, and oral mucosa cells was performed according to our previous research methods (7). Full-length sequencing of the mitochondrial genome was performed simultaneously on samples from the patient, his parents and the elder sister.

## Results

**Figure 1:**
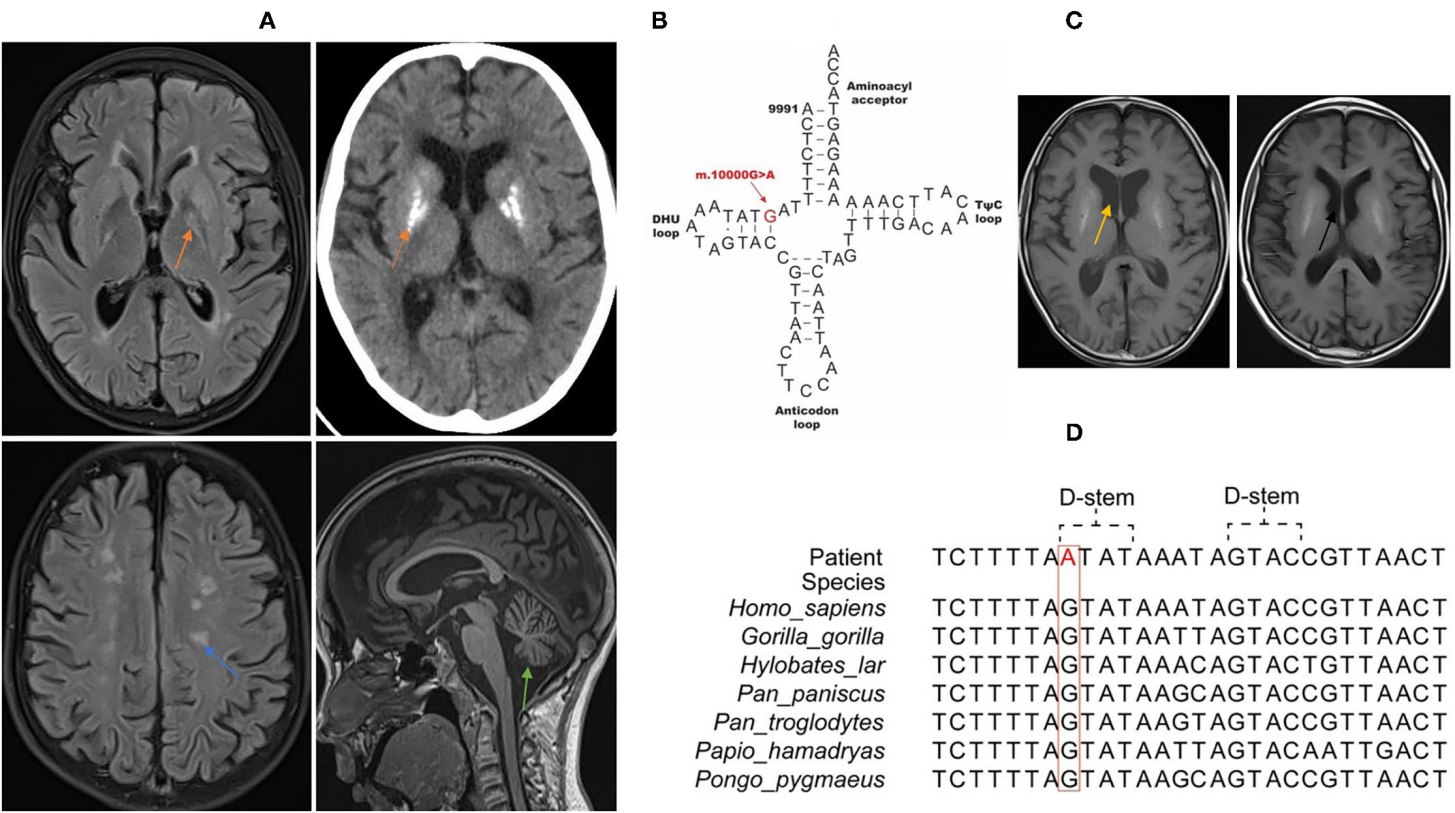
Clinical features and genome profiling reveal a highly conserved, rare, and likely pathogenic *de novo* variant (m.10000G>A) of MT-TG in the 13-year-old patient. **(A)** Brain MRI/CT of the patient. The orange arrow shows basal ganglia calcification. The blue arrow shows multiple lesions in the white matter. The green arrow shows brain stem and cerebellum atrophy. **(B)** Schematic representation of the mitochondrial tRNA^Val^ clover leaf structure and the corresponding location of the novel variation (marked in red) that is confirmed to be a likely pathogenic mt-tRNA^Val^ variant. **(C)** Brain MRI shows that brain atrophy was improved and that the enlarged ventricle seen in previous MRI returned to normal. The yellow arrow shows the enlarged ventricle in acute stage. The black arrow shows the normal ventricle 1 month later after cocktail therapy. **(D)** Phylogenetic conservation of the appropriate regions of multiple mt-tRNA^Val^ gene sequences for m.10000G>A. The highlighted residue is conserved across all the species.

The patient was a 13-year-old boy, and the elder sister and the two non-consanguineous parents were healthy. He presented with frequent vomiting, headache, weakness of the limbs, and hearing loss in several days. Past history included hypothyroidism and exercise intolerance. He was thin and small, and had thick hair on the back and decreased muscle strength of the limbs. Muscle tension was normal, knee reflex was active, and bilateral Babinski's sign was positive. Plasmax lactate was elevated (5.49 mmol/l, reference range 0.44–1.78 mmol/L). White blood cell count, glucose and protein content, microbiological tests, and immune encephalitis-related antibody tests (NMDAR-IgG, AMPA1-IgG, AMPA2-IgG, LGI1-IgG, CASPR2-IgG, GABABR-IgG, MOG-IgG, GFAP-IgG, and AQP4-IgG) on the cerebrospinal fluid were normal. Color Doppler echocardiography examination showed that the aorta and the ascending aorta were widened, and that left ventricular systolic function was normal. Nerve conduction studies and electromyography suggested axonal and demyelination involvement of peripheral neuropathy. Brain MRI (Figure 1A) showed bilateral basal ganglia calcification, multiple lesions in the central white matter, cerebellar atrophy, and enlarged ventricle. Analysis of the next-generation sequencing data (Illumina, United States) of the nuclear genome and entire mitochondrial genome revealed a rare m.10000G>A variant (GenBank reference accession number: NC_012920.1) in MT-TG (Figures 1B,D) that was present at variable heteroplasmy levels across tissue types: 32.7% in the blood, 56.15% urinary in epithelial cells, and 27.3% in oral mucosa cells. No variant was found in the peripheral blood of his mother and sister. There was no pathogenic mutation of nDNA found. He was treated with cocktail therapy (vitamin E, 100 mg bid; vitamin B1, 20 mg tid; vitamin B2, 20 mg tid; vitamin C, 100 mg tid; levocarnitine, 1g bid; coenzyme Q10, 20 mg tid; idebenone, 60 mg tid; arginine, 10 g qd; and intravenous infusion of glucose in the acute stage). The symptoms improved gradually. After one month, he could walk independently. Brain atrophy was improved, and the enlarged ventricle in the MRI returned to normal (Figure 1C). The impairment in hearing was not improved. EMG results still showed peripheral neuropathy involvement.

## Discussion

The clinical features of this patient include hypothyroidism, exercise intolerance, weakness of the limbs, hearing loss, encephalopathy, neuropathy, hyperlactemia, and typical MRI changes. According the mitochondrial diagnostic criteria (MDC) (9), the total MDC score of this patient was 8, namely, muscle weakness (1), abnormal EMG examination (1), multiple system involvement (central nervous system, eye and ear system, short and small, hypothyroidism) (2), multiple examinations of hyperlactatemia (2), and brain MRI showing stroke-like lesions and basal ganglia lesions (2). We also ruled out infection, immunity, and other possible causes, and he could definitely be diagnosed as having mitochondrial disease. Furthermore, at present, studies on other MT-TG variants are mainly focused on functional analysis, and effective treatment methods have not been reported in literature. Our study is the first to report that cocktail therapy is effective for the MT-TG variant. Currently, the treatment of mitochondrial diseases with cocktail therapy to improve mitochondrial energy metabolism is recognized as an effective treatment method. There are corresponding diagnosis and treatment guidelines in China, and a large number of studies have recommended a combination of nutraceuticals (“mitochondrial cocktail”) as a therapy for mitochondrial diseases (1, 10, 11). However, there is still lack of authoritative randomized controlled double-blind studies to confirm the efficacy of this treatment. In our study, after the child was diagnosed with mitochondrial disease, we treated him with cocktail therapy, and his symptoms significantly improved, which was manifested by the fact that one month later, he could walk independently, the brain atrophy improved, and the enlarged ventricle seen in the previous MRI returned to normal. The nDNA of whole exon did not reveal any pathogenic mutation. A novel MT-TG variant was found, which was present at variable heteroplasmy levels across tissue types: 32.7% in the blood, 56.15% in urinary epithelial cells, and 27.3% in oral mucosa cells.

Pathogenic mutations of mtDNA are associated with mitochondrial diseases involving multiple systems and are characterized by heteroplasmy, tissue differences, thresholds, maternal inheritance, and so on. Therefore, making a definitive molecular diagnosis of a mitochondrial disorder is challenging (1, 12). To date, there were only 11 MT-TG variants reported in MITOMAP (http://www.mitomap.org). Among them, only 1 variant, m.10010T>C, is considered “definitely pathogenic.” The known pathogenic variant 10010T>C on the same tRNA was detected in multiple patients, one of which was 15% in skin fibroblasts, 17% in blood and >90% in skeletal muscle (3). In the second patient, it was 89.6% in muscle tissue and 5.4% in blood (4). It was detected in 90.7% of the skeletal muscle of the third patient (5). We suggest that the proportion of this variant in uninvolved tissues such as those of blood and fibroblasts is low, that the proportion in skeletal muscle or brain tissue should be higher, and that the proportion of variants in different tissues varies greatly. Compared to the known pathogenic variant 10010T>C, the novel MT-TG variant 10000G>A has higher mutation load in the peripheral blood. The most common phenotype related to MT-TG is hearing loss (4/11). Other reported phenotypes include chronic intestinal pseudoobstruction with myopathy and ophthalmoplegia, encephalopathy, myopathy, hypertension, progressive encephalopathy, and maternally inherited hypertrophic cardiomyopathy. Several studies reported that mt-tRNA gene variants caused Charcot-Marie-Tooth (13, 14).

In our study, we found a previously unreported MT-TG variant, m.10000G>A, which was located in the DHU loop. In MitoMap, which contains 51,836 full length mitochondrial sequencies, and in gnmoAD V3.1, which contains 56,432 mitochondrial sequencies, this variant's frequencies are 0.004% and 0 respectively. The m.10000 G>A transition dirsupts a Watson-Crick pair in the D stem of the tRNA molecule, which is highly conserved across multiple species, thus impede its stability. The MitoTIP score of m.10000G>A is 20.3833, and Mitomap notation is “likely pathogenic.” The patient was suggested to undergo a muscle or skin biopsy to detect mitochondrial function for further verification, but the parents refused to do this. We also hope that there will be further research in the future to further confirm the pathogenicity of m.10000G>A in terms of mitochondrial function.

We think that this mutation could probably be identified as the pathogenic mutation in this patient because of the following reasons: first, this variant was not listed on the Genome Aggregation Database (https://gnomad.broadinstitute.org/). and his healthy parents and older sister did not carry the *de novo* variant. Second, the patient's phenotype was consistent with mitochondrial disease, and no pathogenic mutation of nDNA was found. Third is the variable heteroplasmy levels across tissue types. Fourth is that hearing loss, encephalopathy, and neuropathy are common clinical features of the reported MT-TG mutant disease (15, 16). Also, in our study, we used a mitochondrial cocktail to treat the patient with MT-TG mutant and obtained improvement in clinical symptoms and brain atrophy. However, randomized controlled large-sample clinical trials are still needed to confirm the efficacy of this treatment regimen. In conclusion, our results added evidence that the *de novo* m.10000G>A variant in the highly conserved sequence of MT-TG appears to suggest a childhood-onset mitochondrial phenotype in the 13-year-old patient, thus broadening the genotypic interpretation of mtDNA-related diseases. Mitochondrial cocktail therapy may be an effective treatment to improve the clinical symptoms of patients with MT-TG mutation.

## Data Availability Statement

The datasets presented in this article are not readily available due to ethical and privacy restrictions. Requests to access the datasets should be directed to the corresponding author.

## Ethics Statement

This study was approved by the Medical Ethics Committee of Hunan Children's Hospital. Written informed consent to participate in this study was provided by the participants' legal guardian/next of kin. Written informed consent was obtained from the individual(s), and minor(s)' legal guardian/next of kin, for the publication of any potentially identifiable images or data included in this article.

## Author Contributions

HY: conducted the literature review and drafted the manuscript. VZ, LA, and SG: make substantial contributions to conception and interpretation of data. LW: revised the manuscript critically and have given final approval of the version to be published. All authors contributed to the article and approved the submitted version.

## Funding

This study was supported by a grant from the National Natural Science Foundation of China (Grant No: 81671297).

## Conflict of Interest

The authors declare that the research was conducted in the absence of any commercial or financial relationships that could be construed as a potential conflict of interest.

## Publisher's Note

All claims expressed in this article are solely those of the authors and do not necessarily represent those of their affiliated organizations, or those of the publisher, the editors and the reviewers. Any product that may be evaluated in this article, or claim that may be made by its manufacturer, is not guaranteed or endorsed by the publisher.

## Footnotes

^1^A Human Mitochondrial Genome Database. http://www.mitomap.org

^2^Genome Aggregation Database. https://gnomad.broadinstitute.org/

^3^ https://www.ncbi.nlm.nih.gov/clinvar/

^4^A Knowledgebase of Human Genes and Genetic Phenotypes. http://www.omim.org
